# Evaluation of DSD training schools organized by cost action BM1303 “DSDnet”

**DOI:** 10.1186/s13023-018-0967-3

**Published:** 2018-12-18

**Authors:** R. Bertalan, A. Lucas-Herald, Z. Kolesinska, M. Berra, Martine Cools, A. Balsamo, O. Hiort

**Affiliations:** 1 First Department of Pediatrics, Semmelweis University, Budapest, Hungary; 2 Developmental Endocrinology Research Group, University of Glasgow, Glasgow, UK; 3 Department of Pediatric Endocrinology and Rheumatology, 2nd Chair of Pediatrics, Poznan University of Medical Sciences, Poznan, Poland; 4 AUSL Modena, Modena, Italy; 5 Department of Paediatric Endocrinology, Ghent University Hospital, University of Ghent, Ghent, Belgium; 6 Pediatric Endocrinology Unit, Reference Center for Rare Endocrine Conditions (CARENDO BO; Endo-ERN), S.Orsola-Malpighi University Hospital, Bologna, Italy; 7 Division of Paediatric Endocrinology and Diabetes, University of Lübeck, Lübeck, Germany

**Keywords:** Differences of sex development network, European Cooperation in Science and Technology, Training school, COST action BM1303

## Abstract

**Background:**

The Differences of Sex Development network (DSDnet) aims to establish interactive relationships between clinicians, scientists, support groups and people with a difference of sex development (DSD) to improve the overall care for people affected by such condition. DSDnet has hosted three Training Schools (TSs) in Ghent, Bologna and Budapest between 2015 and 2017 with the primary purpose of providing multidisciplinary training to young professionals and encouraging ongoing activity in the field of DSD. The aim of our study was to evaluate the success and long-term effect effectiveness of these three TSs.

**Methods and results:**

Eighty-seven trainees (70 women, 17 men) attended one of three TSs. The distribution of trainees according to their professional field was: 47 (54.0%) from Pediatrics/Endocrinology, 13 (14.9%) from Biology/Genetics, 12 (13.8%) from Psychology/Psychiatry and 15 (17.2%) from Surgical Professions. All trainees were asked to complete an evaluation form on the last day of the TS to gain feedback on how to improve the next one. A further survey was sent at the end of 2017 to provide information about the overall long-term impact of the TSs. Seventy-eight (89.7%) trainees completed evaluation forms at the end of the respective TSs. Replies to the subsequent survey were received from 76 (87.4%) of trainees. A total of 72/76 (94.7%) responders reported that they continue to be active in the field of DSD. The vast majority (64/68, 94.1%) reported that the TSs had enlarged their professional networks. Among the 76 respondent trainees, 11.8% (*n* = 9) had applied for a research grant and 10.5% (*n* = 8) had received a fellowship related to DSD since their TS attendance.

**Conclusions:**

According to our results, the majority of TS participants continue to be active in the field of DSD and have enlarged their professional networks following participation at the TS. These findings indicate the need of this type of educational program and justify ongoing efforts to provide postgraduate multidisciplinary training in rare diseases such as DSD.

**Electronic supplementary material:**

The online version of this article (10.1186/s13023-018-0967-3) contains supplementary material, which is available to authorized users.

## Background

Difference (Disorders) of Sex Development (DSD) are rare conditions in which there has been atypical development of the external and/or internal genitalia, the reproductive system and eventually other organs. DSD phenotypes are highly variable and are often associated with lifelong medial problems.

Due to the relatively low frequency of DSDs, there remains limited data regarding optimal management and outcomes for affected individuals and until the formation of international multidisciplinary working groups in the field of DSD around 2000, management has been variable across Europe. Therefore, adequate training of young professionals is needed both for standardized management as well as spreading of expertise. Although many professional organizations such as the European Society of Endocrinology and the European Society of Paediatric Endocrinology organize different types of trainings in the framework of their structural educational program, the DSDnet training school exclusively concentrated on the topic of DSD.

The DSDnet [1] in frame of the Action of COST (European Cooperation in Science and Technology) [2] is an EU-funded program that was created to enable researchers to establish interdisciplinary research networks in Europe and beyond. Funds from COST are available to organize conferences, meetings, training schools, short scientific exchanges or other networking activities in a wide range of scientific topics. The COST Action DSDnet: “A systematic elucidation of differences of sex development (DSD)” was started in 2014 by professionals in the field of DSD, together with representatives from patient organizations. The aims of DSDnet were to obtain new knowledge on the biological pathways of sex development in humans, to standardize management and inform physicians and psychologists caring for people who have a DSD, and to actively collaborate with support groups with the aim of improving patient-centered multidisciplinary care.

To achieve these goals, DSDnet created five working groups, each with different objectives. The working group “Dissemination and Capacity Building” used part of its funds to provide training in DSD for eligible young scientists and therefore organized training schools (TSs) for multidisciplinary healthcare professionals in the field of DSD. Each TS faculty was comprised of approximately 30 students and 10 teachers and the schools took place over five half days (2.5 full days in total). The format of each TS included plenary lectures from the teachers, selected case reports or research presentations by the students delivered in plenary style and interactive training sessions in small groups leaving ample time for discussions.

The Program Organizing Committee included a Scientific Committee, the local organizers and, from the second TS onwards, representatives of the previous TS. The draft of each program was presented, discussed and approved during a preparatory meeting of the Actions Management Committee. The three TSs took place in Ghent, Belgium (2015), Bologna, Italy (2016), and Budapest, Hungary (2017).

The title, topics and trainee target groups for each TS varied as specified below:

1. “Holistic care and research in DSD”: The aim of this first TS was to provide training in various steps of management of DSDs, considering a holistic view on this complex entity. Trainers and trainees were selected from different specialties and professions, ranging from basic and clinical science to psychology and social science. Among the trainers were also a parent and a patient. This meeting provided a range of educational sessions with presentation topics ranging from genetics and development to gender aspects. In addition, there were formal opportunities for trainees to improve their communication skills [3]. The 5th International Symposium on DSD organized by the International Disorders of Sex Development (I-DSD) Registry [4], followed this TS, enabling the extension of the faculty to include trainers from outside the European Union.
2. “People with DSD: holistic care and research through the lifespan**”:** The aim of this TS was to connect young professionals and experts from multidisciplinary backgrounds with each other in an interactive training environment [5]. Attached to this TS, a dedicated workshop was organized for patients and professionals with the aim of jointly developing basic principles of multidisciplinary care [6].
3. “Promoting Research in DSD”: The aim of this TS was to strengthen research collaborations between basic scientists, biologists, geneticists and clinical endocrinologists in the field of DSD [7]. Lecturers gave cutting edge presentations ranging from gonadal dysgenesis and new pathways of steroidogenesis to the research potential of ongoing clinical studies.

To attend the TSs, potential attendees had to complete an application form, which was scored using the following standardized criteria: if they were an early stage researcher (< 8 years post PhD); if they were from an Eastern European country; the quality and the presentation of the application; the overall scientific or clinical merits of the application; the benefit for the Researcher/DSD specific merits and the benefit for the Home Institution. Before the TSs, successful applicants were asked to complete preparatory work including e-learning modules, preliminary reading, a case report and/or a presentation of their own research. The e-learning modules used within the first TS and analysis of their development have been described in two recent papers by Kranenburg et al. [8, 9].

The budgets of the three TSs were 36.970 euros, 23.670 euros and 29.020 euros. Travel grants for trainees were between 150 and 780 euros, depending on the local costs for accommodation and travel distances from the home countries of the trainees to the location of the respective TS. The speakers received reimbursement of their travel expenses according to COST rules for reimbursement [2]. Trainees arriving from countries of the European Union, International Partner Country (IPC), Near Neighboring Country (NNC) and Inclusiveness Target Countries (ITC) were supported according to the COST rules [2].

The primary long-term aim of each TS was to encourage participants to continue and improve involvement in DSD patient care. The second purpose was to promote engagement in the national and international DSD network, for example through participation in the I-DSD registry [4], through grant and fellowship applications in the field of DSD and by becoming active members of the developing European Reference Network for Rare Endocrine Conditions [10].

The aim of this study was to confirm the effectiveness of the TS model and to determine whether the TSs achieved their long term aims.

## Methods

All participants were asked to fill in an anonymous evaluation form about how they had experienced the DSDnet TS on the last day of each meeting. The form was conceived by the Program Organizing Committee of Ghent and included three open questions and 12 responses scored from strongly agree (5) to strongly disagree (1) or from excellent (5) to very poor (1) (Additional file 1: Table S1). Questions were focused particularly on whether the TS met with the expectations of trainees, how they could apply the knowledge gained from the TS in their daily practice, whether the faculty was sufficiently knowledgeable and their overall rating of the TS social program. Following each TS, all the presentations of trainers and case reports of trainees were available on the DSDnet website, with possibilities for further discussion.

A further anonymous survey was sent to all TS trainees at the end of 2017 using the online system Survey Monkey (Table 1). The questionnaire was composed by the authors of the present paper based on Donaldson’s report about the outcome of the European Society for Paediatric Endocrinology (ESPE) Winter Schools [11]. In this survey the participants were first asked about their specialty, job title and training status both at the time of the TS and currently. Participants were also asked about whether they were still active in the field of DSD; whether their professional network was enlarged due to the TS and whether they had applied for or had received any kind of fellowship or grant related to the DSD field since the TS.

**Table 1:** Proofs of the second survey

Questions	Answer choices
Q1. What is your speciality:	o pediatrician/pediatric endocrinologist
	o pediatric/adolescent urologist
	o pediatric/adolescent urologist
	o clinical geneticist
	o researcher (cellular/molecular)
	o clinical psychologist
	o other (please specify) *[free text]*
Q2. Age at the time of TS and Country of Origin	o < 30 yrs
	o 30–35
	o 36–40
	o > 40
	Country of Origin *[free text]*
Q3. TS attended year	o 2015 Ghent
	o 2016 Bologna
	o 2017 Budapest
Q4. Job status at time of TS	*Free text*
Q5. Job status at time of survey	*Free text*
Q6. Are you still active in the field of DSD?	o No
	o Yes
	If no, please explain in brief why not. If yes, please give an example of how you have used the knowledge *[free text]*
Q7. Was your professional network enlarged thanks to the TS? Are you still in contact with:	o Trainees
	o Other members of DSDnet COST Action (specify the specialty or patients’ association) *[free text]*
Q8. Has the DSDnet TS influenced your decision in the direction of your career?	o Yes
	o No
Q9. Have you applied to any kind of grant related to DSD after attending the TS?	o No
	o If yes, please write the name of the grant *[free text]*
Q10. Have you received a fellowship related to DSD after attending the TS?	o No
	o If yes, write the name of the fellowship

The results of these questionnaires were analyzed anonymously by the authors. For the purposes of this study, the term Early Career Investigators refers to researchers within 8 years from the date he/she obtained a PhD/doctorate.

Since the evaluation form and survey were anonymous and involved only healthcare professionals with no clinically sensitive questions, ethical approval was not required.

## Results

In 2015, 2016 and 2017 the number of trainees attending the DSDnet training school was 29 (applied: 64), 29 (applied: 48), 29 (applied: 31), with a total of 87 trainees (one attending twice). Median (range) [interquartile range] age was 33 (24–51) [7], with 15 (17.2%) participants aged 20–30 and 14 (16.1%) aged > 40 years. The gender distribution of the 87 trainees favored women with 70 (80.4%) women and 17 (19.5%) men.

**Fig. 1:**
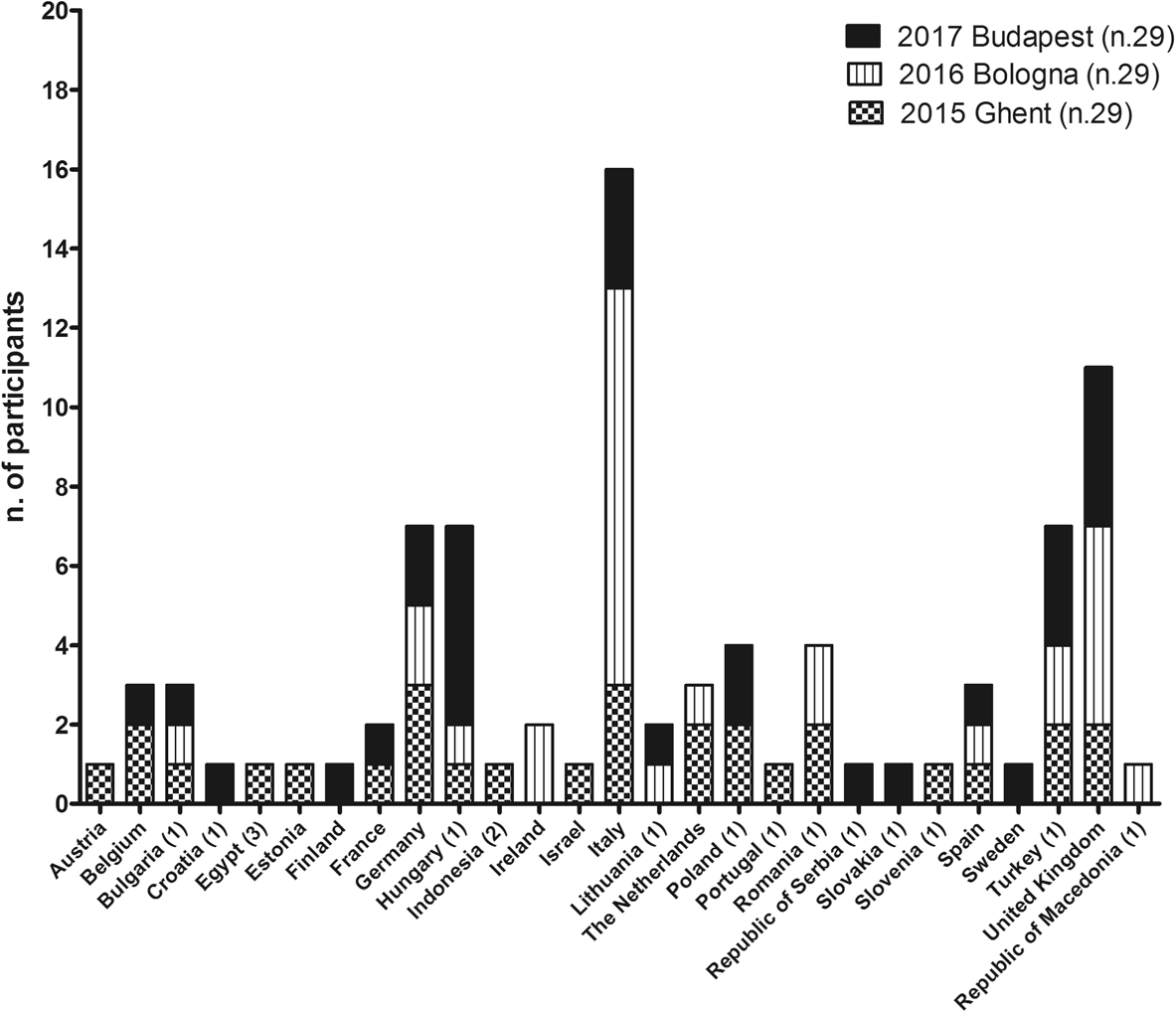
Number of the trainees according to their own Countries. ^1^ Inclusiveness Target Countries (ITC): Bulgaria, Croatia, Hungary, Lithuania, Poland, Portugal, Romania, Slovenia, Slovakia, the former Yugoslav Republic of Macedonia, Republic of Serbia and Turkey. ^2^ International Partner Country (IPC) ^3^ Near Neighboring Country (NNC)

**Fig. 2:**
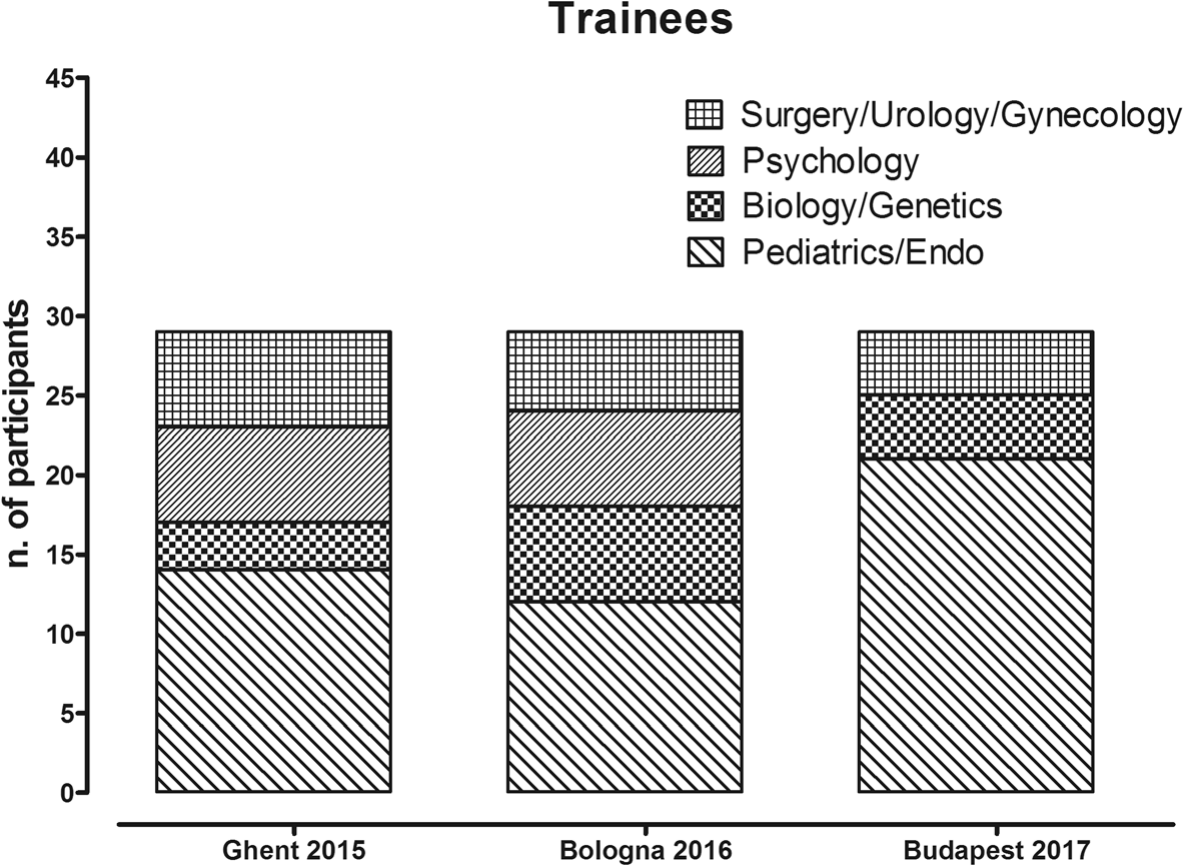
Professional distribution of the trainees

Twenty-five European countries, one International Partner Country (Indonesia) and one Near Neighboring Country (Egypt) were represented (Fig. 1.). There were 30 (34.4%) trainees from Inclusiveness Target Countries and 66 (75.8%) Early Career Investigators present. To minimize the chances for conflict of interest, scholarship awardees were selected by the respective organizing committees according to pre-identified criteria. Applicants and trainees were specialists in the fields of Pediatrics/Endocrinology, Surgery/Urology/Gynecology, Biology/Genetics, Psychology/Psychiatry as shown in Fig. 2.

### Results of the evaluation form

A total of Seventy-eight of 87 (89.7%) trainees completed the evaluation form at the end of each TS of whom 47.4% (37/78) reported that they strongly agreed that the TS had met their expectations; 48.7 (38/78) reported that the TS had met their expectations and 3.8% (3/78) felt neutral about this item. A total of 29/78 (37.1%) strongly agreed that they could use the acquired knowledge from the TS in their daily practice; 43/78 (55.1%) agreed and 6/78 (7.7%) were neutral about this. The faculty were felt to be sufficiently knowledgeable with 72% (56/78) strongly agreeing about this and 25.6% (20/78) agreeing. Overall, the social programs and the TSs as a whole received excellent average ratings. There were no questions scored below 3.

### Results of the survey

The follow-up survey was completed by 76 of the 87 trainees (87.4%), with participants of the three TSs equally represented. There was a 100% response rate to all questions except for one regarding whether the trainees felt their professional network had enlarged after the TS, which was completed by 68 (89.5%) respondents. As the data collection was anonymous we do not have exact information about the nine non-responders.

A total of 94.7% (*n* = 72/76) respondents reported that they *continue to be active in the field of DSD*. Of the 4 participants who were no longer active in this field, the reason reported for discontinuation of DSD activity was incompatibility with their current job role, which had changed since the TS.

**Fig. 3:**
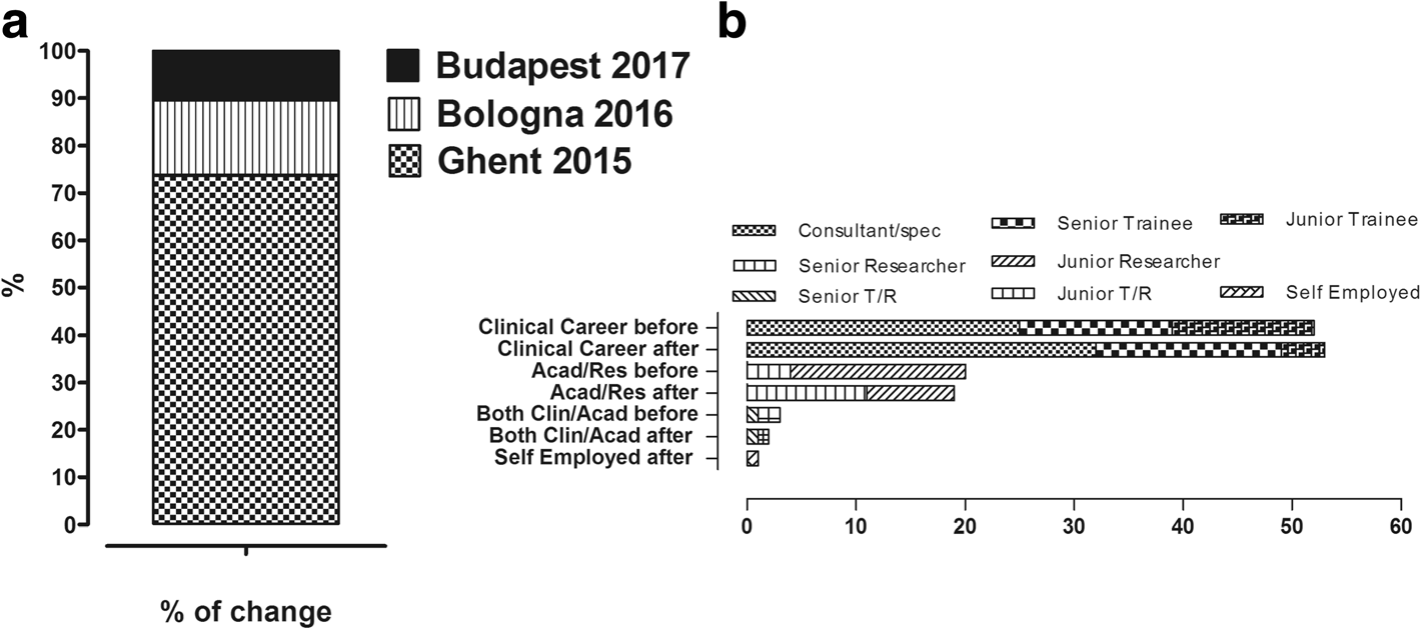
**a** Career advancement (before/after TSs). There were 7 more consultants/specialists, two more senior trainees, 7 more senior researchers, one more junior researcher that combined clinical and academic training. **b** Change of job after the 3rd TS: 25.3% of participants changed job status, of these, around 72% were of the Ghent TS, around 17% of the Bologna TS and 11% of the Budapest TS

The question about *job status* was completed by 76 (87.4%) trainees including one inconclusive response, therefore 75 responses were analyzed. The declared job status had changed after the third TS in 19/75 trainees (25.3%) (Fig. 3a). 18/19 of these trainees had advanced in their career according to their own specialties and the system of their own countries. All of them stayed or moved into the direction of DSD except one junior researcher, who ended an academic career and became self-employed. The distribution of trainees that changed job status after the third TS is shown in Fig. 3b.

**Fig. 4:**
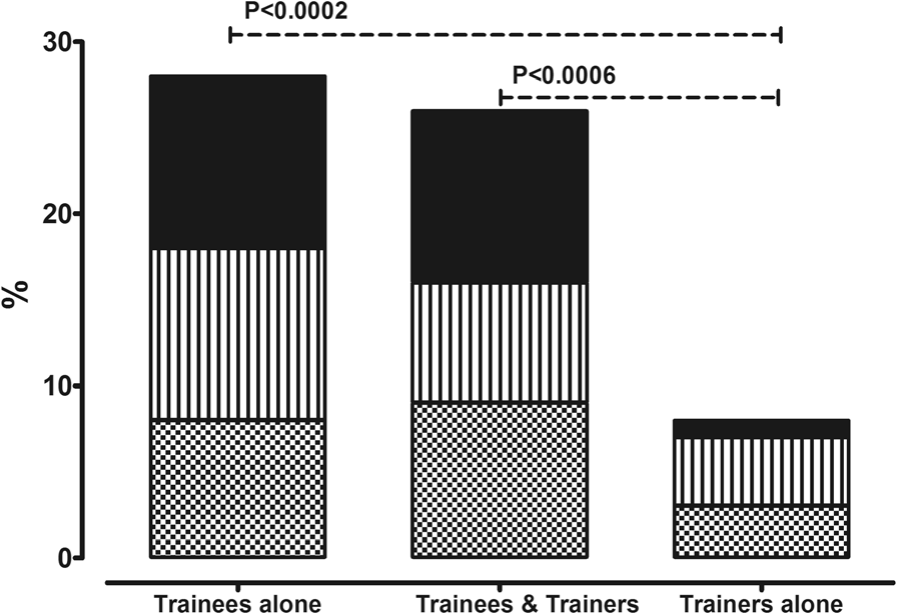
Number of trainees who maintained contact

A total of 68/76 (89.5%) trainees responded to being asked about whether their *professional network had been enlarged* thanks to the TS. Of these, participants were more likely to stay in contact with trainees alone compared to trainers alone (28 vs 8, *p* = 0.0002) or with both trainees and trainers rather than trainers alone (26 vs 8, *p* = 0.0006) (Fig. 4.). A total of 5 (7.3%) (3 from the Ghent TS in 2015 and 2 from the Budapest TS in 2017) reported that they remained in contact with other members of the DSDnet COST action, all of whom also maintained contact with patient associations.

Nine (11.8%) of the TS attendants *applied for a DSD grant* after attendance of a TS. Three of these grants were related to congenital adrenal hyperplasia (CAH), one was claimed within the COST project, one was for DSD related genetics, one was provided by the European Society of Paediatric Endocrinology and no details were supplied regarding the other three. Eight additional trainees (10.5%) reported that they had received a *fellowship related to DSD*: five in the field of pediatric endocrinology, one related to the COST Action, one in the field of pediatric urology and with no further information submitted regarding one. Of the nine applications for grants, six were from students from the 2015 TS, one from the 2016 TS and two from the 2017 TS. The majority of scholarships were therefore awarded to four students who had attended the first TS, with a further two from the 2016 TS and two from the 2017 TS.

## Discussion

Since the beginning of the COST Action “DSDnet” in 2014, three TSs have been organized providing two and a half days of intensive teaching and including free travel and accommodation to 87 trainees from European countries (85), Indonesia (1, IPC), and Egypt (1, NNC). In order to assess the success, usefulness and long-term outcomes of the meetings, we first used an evaluation form, which was completed on the last day of the TS, followed by a survey, which was completed several months after the third TS. It is possible that the initial evaluation form may have been influenced by immediate feelings of gratitude for participation in the TS. This evaluation form was primarily used to gain feedback regarding how to improve the next TS and we received some constructive advice and opinions from the trainees, which were then used for the subsequent TSs. The ratings of the usefulness, the success, the meeting with expectations, the social program and the TS overall were high. No negative responses were received from any trainee. Free suggestions for improvement included: “Lectures could be shorter with more interaction between trainers/trainees”, “Genetics could be simpler”, “More cases with discussion by multidisciplinary experts would be good” and “More interaction with patients would be good”. From the first TS, it became clear that it is difficult to reconcile basic knowledge and expectations from disciplines as different as basis science and psychology. Therefore, the second and third TSs focused on providing holistic care and understanding the biology of DSD respectively. From the second TS onwards, trainees of previous TSs participated in the program organizing committee to ensure a trainee centered focus. Due to the different scopes of the TSs, the professional occupation of trainees varied per training school. For example, only one psychologist applied to participate in the third TS, due to its dedicated topic “Promoting research in DSD”. In the end, this person did not attend as she did not meet the requirements. For this TS, there was also a remarkable decrease in applications. Possibly the proposed team “Promoting research in DSD” has attracted less professionals working in the field of DSD. Alternatively, it is possible that a large proportion of early-career health care workers with an interest in DSD already had the opportunity to participate in one of the previous TSs. Future possibilities for enhanced interaction with patients were explored in a dedicated workshop that took place attached to the 2nd (Bologna) TS [6].

In the second part of our investigation we used a survey, which was sent after the third TS. We hope that this survey therefore gives a more objective evaluation and provides information about the long-term outcome of trainees after having attended one of the TSs. Our survey showed that about a quarter (25.3%) of trainees reported that their job status had changed by the time of the survey. Apart from one trainee who declared termination of an academic career, the others reported career advancements both in clinical and research fields. It seems that the timing of TS attendance might be crucial, as the majority of those with different job status (73.7%) attended the first TS that was held in Ghent in 2015 (Fig. 3a and b).

Given that 94.7% of people enrolled in the survey claim to be still active in the DSD field at the time of the survey, we can assume that trainees who were selected to attend the TSs had a strong motivation to operate in the field and had already made a choice on their career direction prior to TS attendance. Indeed, already working in and being motivated to stay in the DSD field was one of the applied selection criteria for trainees, and our results reflect adequate recruitment of trainees with regard to this criterion. We are convinced that this stringent selection procedure also contributed to the overall success of the TSs and the positive ratings that were received.

In our survey, 89.5% of responders reported that they felt their professional network had enlarged thanks to the TS regardless of which TS was attended. In an increasingly digital age, it is easier than before to maintain contact with colleagues from around the globe and it is hoped that this ongoing contact between TS trainees and trainers will result in productive collaborations, leading to the advancement of care of DSD patients over time. Of note, TS participants were more likely to stay in contact with other trainees rather than with trainers (Fig. 4.). Studies suggest that friendships within organizations such as medical schools or training schools are relatively easily formed due to an increased likelihood of shared common interests [12]. There are several benefits reported in friendships developing between peers working together, including the ability to share knowledge and information [13] and enhanced academic engagement [14]. The high rate of ongoing contact between trainees should therefore be seen as a success of the TS program. This is particularly important for rare disease networks, as peers and partners may not be readily available at their own institution and multicenter collaboration is crucial. In addition, careers involved in rare diseases tend to be hampered by less economic success and also loneliness regarding the field of interest. Therefore, a TS can be highly motivating, and may facilitate successful grant applications and building up one’s personal network.

The numbers of DSD related grants and scholarships obtained after the TSs is limited (11.8 and 9.2%) but the time between the TSs and the survey is short while application times for scholarships and grants are typically much longer. Indeed, as we can see from the results, the majority of the applications for grants (66.7%) and fellowships (50%) were done by students of the first TS in 2015 and it is therefore expected that a number of applications are still to be done.

### General recommendations for the organisation of TSs in the field of rare diseases

- In-depth and interactive discussion of case reports is highly appreciated by participants and a good way to transfer knowledge
- Special attention must be given to make very specialised knowledge accessible for all disciplines involved
- It is important to ensure that the size of groups representing different disciplines is balanced to ensure fruitfull discussions as well as to be able to work in subgroups corresponding to multidisciplinary teams
- More specific topics may reduce the number of applicants
- A social program is an important part of the TS and has to be prepared well also by the organizer
- Evaluation forms provide very useful feedback that can be incorporated in the organisation of subsequent TS on the same topic

### Based on our experience, relevant indicators of long-term success are

- Changes in clinical practice demonstrate the successful transfer of the knowledge during the TS
- Asking about any applications for grants or fellowships in the field of the TS demonstrates the ongoing committment and interest in the fied of the candidates
- Asking about the change of the job status in the direction of the topic of the TS can also present its positive influence.

Retrospectively, we consider the lack of a question assessing the capacity of the TSs to effectively change clinical practice a missed opportunity. For the evaluation of future similar initiatives, we recommend to incorporate at least one question with this purpose, e.g. Did your participation in the TS lead to a change in your clinical practice? If yes, how was this achieved?

## Conclusions

In conclusion, this study highlights the success of the COST Action BM1303“DSDnet” TS model through demonstration of high levels of satisfaction with participation in the TS, high rates of ongoing activity in the field of DSD and a promising DSD related grant and fellowship application rate shortly after termination of the last TS.

## Additional file

Additional file 1: **Table S1.** Evaluation form. (PDF 151 kb)
