# Supplementary material for: Evaluation of DSD training schools organized by cost action BM1303 “DSDnet”
Source: Orphanet J Rare Dis. 2018 Dec 18;13:227. doi: 10.1186/s13023-018-0967-3 (PMC6299629; doi:10.1186/s13023-018-0967-3)
Supplement: Supplementary file 1 — Table S1. Evaluation form. (PDF 151 kb) [file 13023_2018_967_MOESM1_ESM.pdf]

Dear Participant,
Please take a few moments and give us your thoughts on the DSDNet Training School. We would like to learn from your feedback and suggestions.

A. What is your specialty?

I am a
- ❍ ped. endocrinologist
- ❍ (ped./ adolescent) urologist
- ❍ (ped./ adolescent) gynaecologist
- ❍ clinical (ped.) psychologist
- ❍ clinical (ped.) geneticist
- ❍ laboratory (cellular/molecular) researcher
- ❍ other: ……….

B. Please indicate your impressions of the items listed below.

		Strongly Agree	Agree	Neutral	Disagree	Strongly Disagree
1.	The training school met my expectations.	❍	❍	❍	❍	❍
2.	I will be able to apply the knowledge learned in my daily practice.	❍	❍	❍	❍	❍
3.	The content of the lectures was easy to follow.	❍	❍	❍	❍	❍
4.	The faculty was knowledgeable.	❍	❍	❍	❍	❍
5.	The quality of the break out session (molecular genetics) was good.	❍	❍	❍	❍	❍
6.	The quality of the break out session (role play; how to inform) was good	❍	❍	❍	❍	❍
7.	Participation of the participants and interaction with faculty were encouraged	❍	❍	❍	❍	❍
8.	Adequate time was provided for questions and discussion	❍	❍	❍	❍	❍
		Excellent	Good	Average	Poor	Very poor
9.	How do you rate the social program?	❍	❍	❍	❍	❍
10.	How do you rate the training school overall?	❍	❍	❍	❍	❍

C. Which topics or aspects of the training school did you find ***most*** interesting or useful?

1 ______________________________

2 ______________________________

3 ______________________________

D. Which topics or aspects of the training school did you find ***least*** interesting or useful?

1 ______________________________

2 ______________________________

3 ______________________________

E. Please comment on the organization of the Training School (from 1 = insufficient to 5= excellent) 1 2 3 4 5

F. Comments and suggestions (including activities or initiatives you think would be useful for the future)

____

____

____

Thank you
